# An artificial intelligence approach of feature engineering and ensemble methods depicts the rumen microbiome contribution to feed efficiency in dairy cows

**DOI:** 10.1186/s42523-024-00289-5

**Published:** 2024-02-06

**Authors:** Hugo F. Monteiro, Caio C. Figueiredo, Bruna Mion, José Eduardo P. Santos, Rafael S. Bisinotto, Francisco Peñagaricano, Eduardo S. Ribeiro, Mariana N. Marinho, Roney Zimpel, Ana Carolina da Silva, Adeoye Oyebade, Richard R. Lobo, Wilson M. Coelho Jr, Phillip M. G. Peixoto, Maria B. Ugarte Marin, Sebastian G. Umaña-Sedó, Tomás D. G. Rojas, Modesto Elvir-Hernandez, Flávio S. Schenkel, Bart C. Weimer, C. Titus Brown, Ermias Kebreab, Fábio S. Lima

**Affiliations:** 1 Department of Population Health and Reproduction, School of Veterinary Medicine, University of California, 95616 Davis, CA USA; 2 Department of Veterinary Clinical Sciences, Washington State University, Pullman, WA USA; 3 Department of Large Animal Clinical Sciences, University of Florida, Gainesville, FL USA; 4 Department of Animal Biosciences, University of Guelph, Guelph, ON Canada; 5 Department of Animal Sciences, University of Florida, Gainesville, FL USA; 6 Department of Animal and Dairy Sciences, University of Wisconsin, Madison, WI USA; 7 Department of Animal Sciences, College of Agriculture and Life Sciences, University of California, 95616 Davis, CA USA

## Abstract

Genetic selection has remarkably helped U.S. dairy farms to decrease their carbon footprint by more than doubling milk production per cow over time. Despite the environmental and economic benefits of improved feed and milk production efficiency, there is a critical need to explore phenotypical variance for feed utilization to advance the long-term sustainability of dairy farms. Feed is a major expense in dairy operations, and their enteric fermentation is a major source of greenhouse gases in agriculture. The challenges to expanding the phenotypic database, especially for feed efficiency predictions, and the lack of understanding of its drivers limit its utilization. Herein, we leveraged an artificial intelligence approach with feature engineering and ensemble methods to explore the predictive power of the rumen microbiome for feed and milk production efficiency traits, as rumen microbes play a central role in physiological responses in dairy cows. The novel ensemble method allowed to further identify key microbes linked to the efficiency measures. We used a population of 454 genotyped Holstein cows in the U.S. and Canada with individually measured feed and milk production efficiency phenotypes. The study underscored that the rumen microbiome is a major driver of residual feed intake (**RFI**), the most robust feed efficiency measure evaluated in the study, accounting for 36% of its variation. Further analyses showed that several alpha-diversity metrics were lower in more feed-efficient cows. For RFI, *[Ruminococcus] gauvreauii group* was the only genus positively associated with an improved feed efficiency status while seven other taxa were associated with inefficiency. The study also highlights that the rumen microbiome is pivotal for the unexplained variance in milk fat and protein production efficiency. Estimation of the carbon footprint of these cows shows that selection for better RFI could reduce up to 5 kg of diet consumed per cow daily, potentially reducing up to 37.5% of CH_4_. These findings shed light that the integration of artificial intelligence approaches, microbiology, and ruminant nutrition can be a path to further advance our understanding of the rumen microbiome on nutrient requirements and lactation performance of dairy cows to support the long-term sustainability of the dairy community.

**Supplementary Information:**

The online version contains supplementary material available at 10.1186/s42523-024-00289-5.

## Background

Over the last 60 years, the use of genetic selection has helped more than doubling the milk produced per cow and contributed to improving dairy farms’ sustainability and environmental stewardship [1–4]. Genetically selecting cows that can decrease the dairy carbon footprint has been one of the most promising approaches to date among almost 100 strategies tested to reduce global warming and improve farm sustainability [5]. Initial studies show that retaining in the herd cows that are more efficient in converting feed into milk (e.g., feed efficient) could reduce total methane (**CH**_**4**_) emissions from 11 to 26% in 10 years [3]. In another study that measured the potential CH_4_ reduction showed that including feed efficiency in breeding programs could reduce 0.41-0.43 g of CH_4_ per kg of milk produced (e.g., ECM) [6]. However, even with the success of genomic selection and other feasible approaches, achieving the target of limiting global warming to 1.5 °C by 2030 established by the Paris Agreement will only be possible with the full global implementation of these strategies [5, 7, 8]. Even then, the 1.5 °C for 2050 is not expected to be met due to the offsetting effects of rising CH_4_ emissions from increased meat and milk demands for human consumption [5]. Therefore, a parallel further development of effective mitigation approaches tied with a continuous optimization of animal productivity that contributes to achieving the established climate targets and ensuring the sustainability of the dairy industry and global human population is urgently needed.

Although several feed and milk production efficiency traits have been explored for genomic selection, such as gross feed efficiency measures (kg milk produced/kg feed intake), residual feed intake (**RFI**) is a major contributor to the net merit of dairy cows and the feed efficiency metric used to calculate other traits, such as feed saved [9–11]. The importance of RFI relies on the robustness of the trait that corrects the gross feed efficiency to body weight, body energy changes over time, and parity of the cow, yielding an efficiency measure that more realistic represents the true feed efficiency of a cow [12]. In terms of RFI applicability, the trait has been reported to have heritability and repeatability across lactation of 0.14 and 0.24, respectively, in U.S. Holstein cows, which suggests that there is potential for genetic selection [11]. However, the difficulty in collecting overall feed efficiency phenotypes (i.e., the necessity to measure daily individual feed intake) is exacerbated for RFI that also requires individual body weight and body energy changes over time, and so limits the speed by which the reference population can be expanded for genomic predictions and poses a challenge in the selection of feed-efficient cows [13–15]. Thus, tracing all sources of variation that can be used to improve RFI prediction and more quickly expand the reference population is paramount to reliably retain in the herd highly feed-efficient cows or even improve the feed efficiency status of less efficient ones [7].

**Fig. 1:**
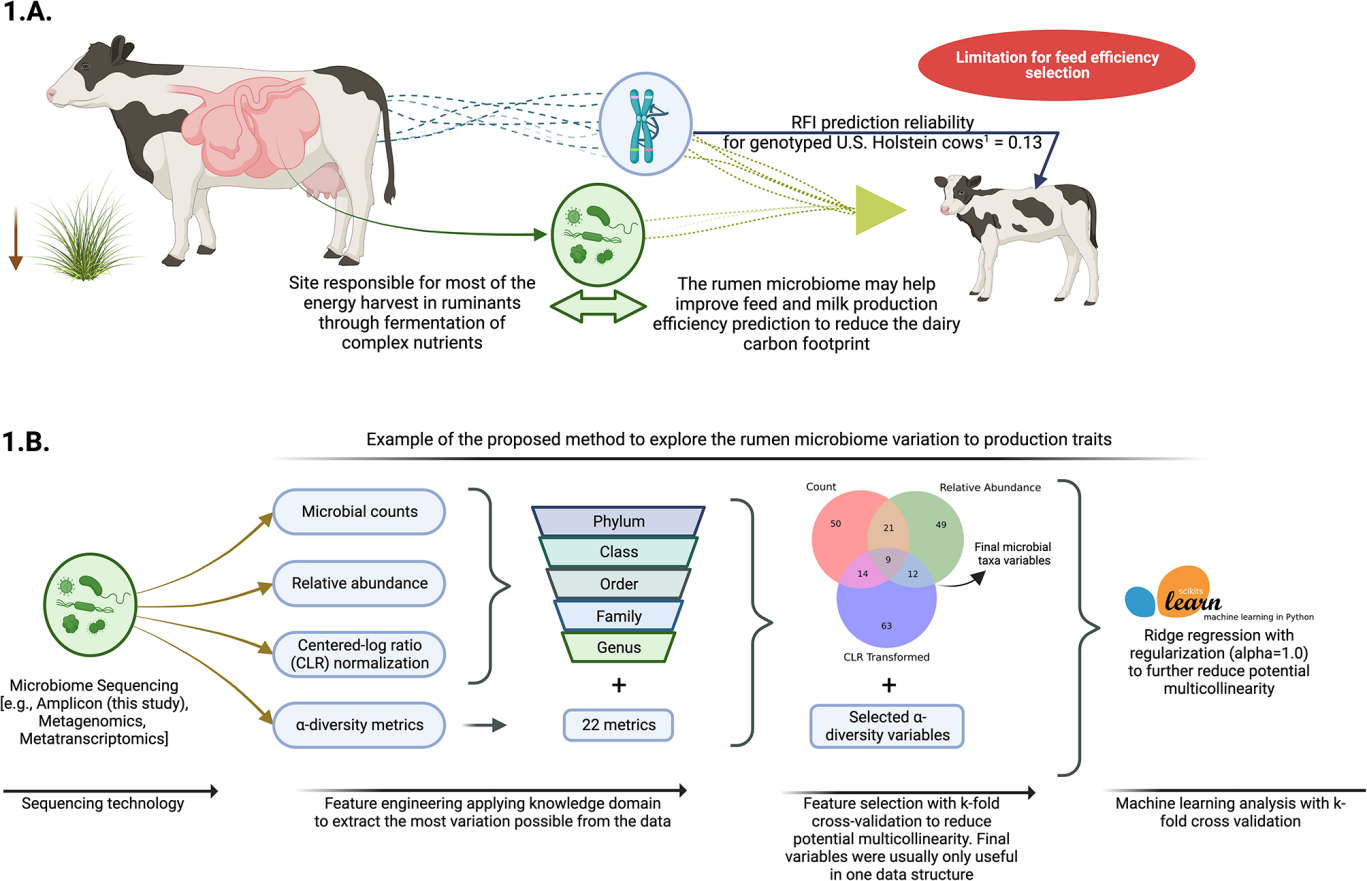
The rumen microbiome signature as a path for predicting feed efficiency and improving the selection of dairy cows with a lower carbon footprint. **1.A.** Mechanism of how the prediction and selection for feed efficiency could be improved by adding the rumen microbiome signature for a trait or potential biomarkers. The illustration depicts the reliability issue of residual feed intake (**RFI**), one of the main feed efficiency traits under investigation. ^1^Citation: B. Li, et al. Genomic prediction of residual feed intake in US Holstein dairy cattle. *J. Dairy Sci.* 103 (3): 2477–2486, 10.3168/jds.2019-17332 (2020); **1.B.** Scheme depicting an example of the proposed method to explore the potential variation from the rumen microbiome composition in production traits. The prediction was based on feature engineering and taking into account each taxonomy level and data structure may have additional explainability to model

Amongst RFI sources of variation, the rumen microbiome plays a key role in converting plant polysaccharides into energy available to the animal, which accounts for up to 70% of cattle’s total caloric requirements [16–18]. However, ruminal fermentation of dietary nutrients also has some inefficiencies, and waste products such as ammonia, CO_2_, and CH_4_ are produced, which only the latter can represent 5.7 to 7.5% of the cow’s gross energy intake that is wasted [17, 19, 20]. These facts have made the rumen microbiome modulation a focal point for efforts to optimize dietary nutrient conversion into milk [16–18, 21–23]. Notably, cows that are naturally more feed efficient have been reported to potentially harvest more energy from the same nutrient intake [4, 21], suggesting their rumen microbiome differs and together with an improved host metabolic activity, these could be key components of improved feed efficiency. Several studies support the hypothesis the rumen microbiome composition and activity of more feed-efficient dairy cattle differ from less efficient ones [22–25], suggesting this component of cow’s phenotypic variation should also be considered in RFI predictions. Studies suggesting the existence of heritable rumen microbes controlled by the host genetics [22, 26] indicate that predicting dairy cows with more efficient rumen microbiomes or identifying ruminal markers associated with RFI could contribute to improve the reference population challenges faced in genetic selection. However, quantifying the RFI variation from the rumen microbiome’s count-compositional data remains a formidable task since pioneering studies in microbiome predictions [27], given its high-dimensional and complex nature [28]. In this context, artificial intelligence (**AI**) such as machine learning algorithms, is uniquely equipped to manage the inherent multicollinearity of such datasets and discern intricate patterns from microbial compositions [28, 29]. Furthermore, harnessing AI approaches, such as feature engineering and ensemble methods, can be a first step to fully explore the potential variation in RFI coming from the microbiome. Thus, applying AI to microbiome datasets could pave the way for the exploration of biomarkers associated with such variation and refine genomic prediction for feed efficiency Fig. 1.

Therefore, we hypothesized that exploring the rumen microbiome composition with AI approaches such as feature engineering, that applies knowledge domain from bioinformatics to extract more information from variables, could be a path to quantify the feed and milk production efficiency variation associated with the rumen microbiome composition. We further hypothesized that there is a portion of variation in gross feed efficiency (a.k.a. milk production efficiency) that is attributed to some rumen microbes independent of feed intake and milk production levels, body weight, body energy changes, and parity. Our objectives were to use feature engineering and develop a novel network analysis of ensemble methods to explore the rumen microbiome composition contribution to these feed and milk production traits in a genotyped population of Holstein cows (*n* = 454) in the U.S. and Canada. Consequently, specific rumen microbes that support the core microbial community structure contributing to RFI and milk production efficiency of dairy cows were highlighted. A final comparison of the potential selection based on measured RFI, microbiome predicted RFI, and genomic predicted transmitting ability (PTA) RFI was performed to highlight the potential reduction in the carbon footprint of dairy cows through RFI selection. The findings from the study advance our understanding of the rumen microbiome’s contribution to predicting feed and milk production efficiency in dairy cows and offer a new approach to exploring microbiome contribution to related productive traits.

## Methods

This experiment was conducted following all guidelines approved by the Institute of Animal Care and Use Committee from the University of California, Davis (protocol #21,864), University of Florida (protocol #201,910,673), and University of Guelph-Canada (Animal Utilization Protocol #4064).

### Experimental design and data collection

This multi-site prospective study focused on lactating Holstein cows (*n* = 454) and was conducted in two research centers. The cows in the study were housed at the Dairy Unit (Alachua, FL, United States; *n* = 238) from the University of Florida (**U.S.**) and the Ontario Dairy Research Centre (Elora, ON, Canada; *n* = 216) of the University of Guelph, Canada (**Canada**). This study was conducted in parallel to 6 experiments that totaled 19 treatments, which were accounted in our statistical models. Eligibility criteria for enrolled cows included no history of abortions, twins, cesarean sections, or antimicrobial therapy within seven days preceding rumen sampling. Overall, this study had 221 primiparous and 233 multiparous cows that met these criteria. Experimental diets and their chemical composition are reported in Table 1.

**Table 1:** Experimental summary and chemical composition of the diets used in the study (% of DM unless otherwise stated)^1^

			United States					
*Item*	Mean ± SD	CAN1^2^	UF1^3^	UF2^4^	UF3^5^	UF4^6^	UF5^4^	
*n*	454 (total)	216	35	40	51	12	100	
Rumen sampling, *DIM*	62 ± 3	64	60	60	60	66	60	
First day, *DIM*	56 ± 15	42	50	50	50	85	61	
Last day, *DIM*	105 ± 12	91	100	105	100	125	110	
Total collection days	50 ± 3	49	51	56	51	40	49	
DMI, *kg/d*	22.6 ± 2.60	24.2	22.2	23.9	20.9	21.3	23.1	
Milk production, *kg/d*	40.4 ± 6.80	40.5	40.8	40.3	34.7	42.2	43.8	
NESec, *Mcal/d*	27.1 ± 4.20	29.1	26.6	27.1	25.4	25.8	28.6	
BW^0.75^, *kg*	127 ± 9.70	132	121	137	119	122	134	
BEC, *kg/d*	2.60 ± 3.20	2.25	2.26	4.51	2.38	0.66	3.25	
*Chemical composition*								
OM	92.7 ± 0.61	93.4	92.1	92.5	92.4	93.7	92.8	
CP	17.0 ± 0.88	15.8	16.8	17.7	18.4	16.3	16.9	
RDP^1^	10.9 ± 0.47	10.7	11.9	10.1	11.0	11.4	11.4	
RUP^1^	5.22 ± 0.13	5.10	5.40	4.90	5.20	5.00	5.20	
NDF	28.6 ± 4.07	29.3	29.8	34.2	23.5	26.8	25.3	
Forage NDF	23.0 ± 2.63	25.6	18.4	19.3	21.6	18.2	22.5	
Starch	30.5 ± 1.61	27.1	31.1	27.9	31.7	31.8	31.5	
ADF	16.0 ± 3.02	19.4	16.6	18.3	15.7	10.7	15.1	
NFC	43.1 ± 5.37	45.1	41.4	33.4	45.7	48.6	46.1	
Ether extract	4.64 ± 1.59	3.63	4.10	7.27	5.44	4.26	4.26	
NEL, *Mcal/kg of DM*	1.69 ± 0.03	1.68	1.67	1.74	1.70	1.73	1.65	

^1^DIM = days in milk, DMI = dry matter intake, NESec = net energy secreted in milk, BW = body weight, BEC = body energy changes, OM = organic matter, CP = crude protein, RDP = rumen degraded protein, RUP = rumen undegraded protein, NDF = neutral detergent fiber, ADF = acid detergent fiber, NFC = non-fibrous carbohydrates, NEL = net energy required of lactation, kg = kilograms, and Mcal = megacalories, SD = standard deviation, CAN = Canada, UF = University of Florida (U.S.A.)

^2^Mion et al. (2023), DOI: 10.1093/jas/skad041

^3^Zimpel et al. (2021), DOI: 10.3168/jds.2021-20486

^4^Unpublished. Ingredient composition can be accessed in Supplementary Table 1

^5^Oyebade et al. (2023), DOI: 10.3168/jds.2022-22898

^6^Lobo et al. (2023), DOI: 10.3168/jds.2022-22583

All cows had their individual dry matter intake (**DMI**), body weight (**BW**), and production data recorded daily on average between 56 (SD ± 15) to 105 (SD ± 12) days in milk, following the principal of RFI repeatability reported by Connor et al. [30]. The DMI was measured in U.S. cows through individual feeding gates (Calan Broadbent Feeding System, American Calan Inc., Northwood, NH) and in Canada through automated feed bins (Insentec B.V., Hokofarm Group, Emmeloord, AX, Netherlands). Daily DMI was measured as the weight differences between the amounts of offered total-mixed ration and refusals multiplied by the dry matter content of the diet. The BW was recorded twice daily using a walk-through scale (AfiWeigh, SAE Afikim, Israel in the Florida herd; and DeLaval, Tumba, Sweden in the Canada herd) right after each milking, from which a mean daily metabolic BW (**MBW**; BW^0.75^) was calculated and used for subsequent calculations. Body condition score (**BCS**) was assessed weekly by trained evaluators following a 1–5 scale with increments of 0.25 units, as described in the Elanco BCS chart (Elanco Animal Health, 2009). Body energy changes (**BEC**) were calculated according to the following equation of the National Research Council (NRC), Nutrient Requirements of Dairy Cattle [31]:

BEC = [2.88 + (1.036 x BCS week)] x BW change (kg/d).

Cows were milked twice daily, with milk yield being recorded using electronic milk flow meters (AfiFlo, SAE Afikim, in the United States herd; and DeLaval, in the Canada herd). Milk samples were collected once (Canada) or twice (U.S.) weekly from both milking times for the analyses of milk fat, true protein, and lactose. Milk composition for U.S. cows was analyzed at the Southeast Milk Inc. laboratory (Belleview, FL) that is part of the Dairy Herd Improvement Association, and in Canada at the Lactanet Guelph Analysis Center laboratory (Guelph, ON). The yields of milk fat, true protein, and lactose from each milking time were used to calculate the daily milk components’ yield from each cow. Unless otherwise stated, milk production is presented throughout the manuscript as amount of NE secreted in milk or NESec (Mcal/d), which was calculated based on the energy values of milk fat, protein, and lactose according to the NRC equation:

NE secreted in milk (NESec) = [(9.29 x kg fat) + (5.47 x kg protein) + (3.95 x kg lactose)]

### Feed efficiency calculations in linear mixed-models

The main feed efficiency measure of the study (RFI) was calculated by fitting linear mixed-effect model using the MIXED procedure in SAS (SAS Institute Inc.) and assessing the difference between their measured DMI and the predicted one [12, 32]. The prediction of DMI was performed for each cow after fitting a linear mixed-effect model using the MIXED procedure in SAS (SAS Institute Inc.) adjusting for the cohort and accounting for the feed intake and major energy sinks in a lactating cow, which are NESec, MBW, and BEC. The linear model included the fixed effects of parity, NESec, MBW, and BEC, and the random effects of treatment nested within the experiment (cohort) and the residual error term, which is the RFI. A negative RFI value identified cows that were more efficient as they had lower DMI than predicted, whereas a positive RFI value identified cows that were less efficient because they had greater DMI than predicted. Statistical significance was declared when *P* ≤ 0.05. The total coefficient of determination (**R**^**2**^) of the regression for DMI was 0.81. Consequently, the residuals of the model (RFI) accounted for 19% of the variation in DMI, which was later used for microbiome predictions. Based on Type III SS in order to evaluate the importance of each predictor, the R^2^ for parity (R^2^ = 0.02), MBW (R^2^ = 0.12), BEC (R^2^ = 0.05), NESec (R^2^ = 0.39), and treatment within experiment (R^2^ = 0.07) are presented in Table 2.

**Table 2:** Results from a linear mixed model for dry matter intake and gross feed efficiency traits in 454 lactating Holstein cows in the US and Canada. Individual parameters were tested using Type III sum of squares

Item	R^2^	Estimate	SE^1^	*P*-value
**Dry matter intake, kg/d**				
*Parity*^2^	0.02	0.87	0.22	< 0.001
*MBW, kg*	0.12	0.09	0.01	< 0.001
*BEC, Mcal/d*	0.05	0.17	0.02	< 0.001
*NESec, Mcal/d*	0.39	0.37	0.02	< 0.001
*Treatment (random effect)*	0.07			
*Total R*^*2*^*of the regression*^*3*^	0.81			
*Residual feed intake (RFI)*^*3*^	0.19			
**Milk fat efficiency, g/kg DMI**				
*Parity*^2^	0.01	-3.95	0.64	< 0.001
*MBW*	0.01	0.05	0.03	0.04
*BEC*	0.00	-0.03	0.07	0.63
*DMI*	0.18	-2.97	0.13	< 0.001
*NESec*	0.58	2.71	0.07	< 0.001
*Treatment (random effect)*	0.07			
*Total R*^*2*^*of the regression*^*3*^	0.84			
*Residual MFE*^*3*^	0.16			
**Milk protein efficiency, g/kg DMI**				
*Parity*^2^	0.02	2.84	0.49	< 0.001
*MBW*	0.00	-0.03	0.02	0.13
*BEC*	0.00	0.01	0.05	0.82
*DMI*	0.20	-1.80	0.10	< 0.001
*NESec*	0.41	1.30	0.05	< 0.001
*Treatment (random effect)*	0.07			
*Total R*^*2*^*of the regression*^*3*^	0.69			
*Residual MPE*^*3*^	0.31			

^1^Standard error of the estimate

^2^Primiparous were used as the reference for the parameter parity, meaning the estimate is in regard to the multiparous Holstein cow effect

^3^These R^2^ were calculated based on the REML default PROC MIXED model in SAS 9.4 and used to derive the residual of feed intake (RFI), residual of MFE, and residual of MPE. These values are not the sum of each parameter R^2^ reported in this table as those are based on the Type III sum of squares method chosen for hypothesis testing on each parameter

Beyond RFI measurement, as a feed efficiency trait, this study also focuses on the efficiency of producing milk fat and protein (**MFE** and **MPE** in g/kg d, respectively), as gross feed efficiency measures (Table 2). These were calculated as the ratio between product and feed intake:

$$ \left(1\right)\,MFE=\frac{\text{g}\,\text{o}\text{f}\,\text{m}\text{i}\text{l}\text{k}\,\text{f}\text{a}\text{t}\,\text{p}\text{r}\text{o}\text{d}\text{u}\text{c}\text{e}\text{d}}{\text{k}\text{g}\,\text{o}\text{f}\,\text{D}\text{M}\text{I}},$$

and

$$ \left(2\right)\,MPE\,=\frac{\text{g}\,\text{o}\text{f}\,\text{m}\text{i}\text{l}\text{k}\,\text{p}\text{r}\text{o}\text{t}\text{e}\text{i}\text{n}\,\text{p}\text{r}\text{o}\text{d}\text{u}\text{c}\text{e}\text{d}}{\text{k}\text{g}\,\text{o}\text{f}\,\text{D}\text{M}\text{I}}$$

and later fitted in a similar linear mixed model as in the previously described model for RFI to assess the portion of variation in gross feed efficiency that is not attributed to intake and production levels, body weight, body energy changes, and parity beyond of previous treatment. Similar to how the digestibility of the diet could affect gross feed efficiency metrics, we explored how the 16 S rRNA rumen microbial composition would affect these efficiency metrics as well. The model contained the fixed effects of parity, DMI, NESec, MBW, and BEC and the random effect of treatment nested within experiment as well as the residual error on the traits of interest. The R^2^ of the full model [MFE (R^2^ = 0.84) and MPE (R^2^ = 0.69)] and the importance of each parameter to the predictions are also reported in Table 2. The NESec accounted for 58% of the variation on MFE while accounting for 41% on MPE, which is a similar variation to that observed on the predicted DMI for RFI. A concern on the use of gross efficiency measures is the fact that they do not account for body size and composition, which is relevant for maintenance requirements and nutrient or energy partitioning [13]. In this study, little to no effect was observed from MBW and BEC on MFE and MPE, respectively, suggesting that in certain conditions these gross efficiency measures can be used as a proxy of efficiency for these cows. The residual MFE (R^2^ = 0.16) and residual MPE (R^2^ = 0.31) were later also used for subsequent microbiome predictions, as this portion of variation in these efficiency metrics would not be affected by the levels of feed intake, energy outputs, parity, and previous treatments these cows were undergoing. In these cases of residual milk production efficiency, a cow with greater residual MFE or MPE represents a cow that was more efficient than the parameters in the model could predict.

### Rumen content collection

Rumen contents were collected from all 454 cows in the study at 62 (SD ± 3) days in milk. An oro-esophageal tubing procedure was used at 2 to 6 h after the morning feeding time, as described by Monteiro, et al. [25] and Cunha, et al. [33]. The procedure consisted of a vacuum pump equipped with a glass container that was connected to a probe of 200 cm long and 2.5 cm of diameter. The probe was carefully inserted orally through the esophagus until it reached the rumen compartment. The first 150 mL of rumen contents were discarded, and the subsequent 250 mL were immediately separated for pH measurement and storage in 15 mL conical tubes for further microbiome analysis. Rumen content pH was measured using a portable pH meter and samples were snap-frozen in liquid nitrogen to preserve microbial DNA. Later, rumen samples were transferred to a -80 °C freezer until further microbial DNA extraction and sequencing.

### DNA extraction, library preparation, and sequencing

The DNA extraction and library preparation were all processed through a 96-channel portable robot pipette to improve pipetting precision. On the day of DNA extraction, rumen content samples were thawed in ice, and DNA was extracted using a Mag-Bind Universal pathogen 96 Kit (Omega Bio-Tek Norcross, GA) according to manufacturer instructions. Library preparation was performed according to the standard protocol from the Earth Microbiome Project [34]. Briefly, the goal was to amplify prokaryotes (mostly bacteria) by targeting the V4 region of the 16 S rDNA of the isolated microbial genomic DNA. The forward and reverse primers used were GTGYCAGCMGCCGCGGTAA (515 F - Parada) and GGACTACNVGGGTWTCTAAT (806R - Apprill), respectively, as described by Parada et al. [35] and Apprill et al. [36]. After DNA amplification, the presence and size of amplicons were verified through gel electrophoresis using a 1.2% (wt/vol) agarose gel stained with 0.5 mg/mL ethidium bromide. Purification of amplified DNA was performed through magnetic Mag-Bind TotalPure Next Generation Sequencing (Omega Bio-Tek, Norcross, GA, USA) following manufacturer instructions. The DNA concentration and purity were assessed through spectrophotometry, considering that the concentration of pure DNA with an A_260_ of 1.0 was 50 µg/ mL. Then, DNA samples were diluted to the same concentration using ultrapure distilled water, and an equal volume of each sample were pooled together for sequencing. A final accurate and precise DNA quantification was performed in a Qubit® fluorometric machine. For sequencing, the pooled library was diluted to 4 n*M*, denatured, and combined with a PhiX Control 3 following the MiSeq System Denature and Dilute Libraries Guide (Illumina, San Diego, CA, USA). A MiSeq Reagent Kit v2 of 300 cycles (Illumina, San Diego, CA, USA) was used in an Illumina MiSeq platform set for a 16 S Metagenomics Workflow. All sequences were deposited in the Sequence Read Archive of the National Center for Biotechnology Information under the BioProject accession number PRJNA962991.

### Bioinformatic analysis of 16S rRNA sequences

The first step in our analysis was the creation of metadata containing all information from each cow (*n* = 454). These cows were within the eligibility criteria for enrollment described earlier, they were not repeated in more than one lactation, and they had enough sequencing depth based on the rarefaction curve (> 3000). Then, amplicon sequences were processed in R through the *dada2* pipeline version 1.8, first described by Callahan, et al. [37]. Briefly, denoising analysis was performed by demultiplexing sequencing reads, and inspecting them for quality and errors. Based on that, sequences were trimmed and filtered, chimeras were removed, and an amplicon sequence variant (**ASV**) table was created. Taxonomy assignment was performed using the 16 S rRNA SILVA v138 database (date accessed: 26-Jan-2022. https://www.arb-silva.de/documentation/release-138/) up to the genus level [38–40]. Using the phyloseq package [41], total ASVs representing microbial counts were split into taxonomy levels (phylum, class, order, family, and genus). Two normalization methods were performed in the microbial counts within each taxonomy level. The first normalization was to relative abundance, which represented the direct proportion of microorganisms to the total number of reads in the sample (0 to 100%). The second normalization was to centered-log ratio (**CLR**), which first applies a log transformation to the ratio between the microbial taxa counts and the geometric mean of microbial counts in a sample, and then centers it by subtracting them from the mean of the natural log values for each sample (-15 to + 15), as first described by Aitchison [42] and more recently reported to capture more patterns in compositional-count datasets [43–45]. The datasets containing microbial counts, relative abundances, and CLR normalized data were each used in the model in order to identify all potential patterns in the contribution of microorganisms to the production traits evaluated in the study. Twenty-two alpha-diversity indexes (observed sequences, chao1, diversity_inverse_simpson, diversity_gini_simpson, diversity_shannon, diversity_fisher, diversity_coverage, evenness_camargo, evenness_pielou, evenness_simpson, evenness_evar, evenness_bulla, dominance_dbp, dominance_dmn, dominance_absolute, dominance_relative, dominance_simpson, dominance_core_abundance, dominance_gini, rarity_log_modulo_skewness, rarity_low_abundance, rarity_rare_abundance) were calculated to further extract all possible variation in the data through knowledge domain using the *microbiome* and *vegan* packages in R [46, 47], which were subsequently added to the final dataset.

### Machine learning with feature engineering

All analyses were performed in Python using the packages NumPy, Seaborn, Pandas, Scikit-learn, mlxtend, TensorFlow, and Keras. Several methods were investigated for the predictions of production traits using the rumen microbiome, such as traditional linear regression models, dimension reduction analyses, machine learning algorithms, and deep neural networks. Similar to Wallace et al. [22], a Ridge regression had the best fit among evaluated methods. For feature engineering, all taxonomy levels (phylum, class, order, family, and genus) in three data structures (count, relative abundance, and CLR), and alpha-diversity indexes were used for variable selection, totaling 2,194 ASVs. In order to better deal with multicollinearity and improve regularization, the model was fitted with a feature selection approach based on the lowest mean squared error (**MSE**) loss function in a 10-fold cross-validation. A final model was considered to include the most associated variables with the production trait of the study. The final model coefficient of determination (**R**^**2**^), MSE, and root of the MSE (**RMSE**) were calculated based on the validation sets from the cross-validation. Because the main evaluated efficiency metrics were based on residuals, those values were split between positive and negative, and a confusion matrix was created following their predictions. Whenever the efficiency metric was in the same status of the observed value (observed positive or negative phenotype), observations were assigned a true positive or negative category. The opposite was also used to create false positive and false negative predicted values.

### Ensemble method of differential abundance analyses

To avoid the previously reported bias of lack of consistency across differential abundance analysis (**DAA**) methods [48], we aimed to build an ensemble method to report microbial taxa that would be consistently different across several approaches [49]. Given the large number of experimental units in this study, the ensemble method could be built in two parts. The first and unique part for the large number of experimental units, consisted of using all observations in the study (*n* = 454) to have a broader overview of microbial taxa importance to the trait of interest. Thus, a linear regression model containing all the engineered variables were fit in a STEPWISE selection (*P* ≤ 0.05; and lowest AICC) to determine the variables that were associated with the total variation that could be explained by the microbiome on the respective traits. Still in the first part, the taxa deemed significant for our AI predictions were extracted and also fed to the ensemble analysis. The second part and more commonly done for DAA analysis, consisted of comparing phenotype extremes within the study population. For that, the cows within the extremes of each trait of interest (most: *n* = 50; least: *n* = 50 cows) were selected for microbial community composition differences. Four methods were used for these analysis: ALDEx2 [50–52], ANCOM-BC [53], MaAslin2 [54], and LinDA [55]. The first three were some of the most consistent methods and with low false discovery rate across different datasets tested in Nearing et al. [48] while LinDA is a newer method reported to have potentially even lower false discovery rate compared to the other three methods but that can capture compositional bias using the mode of all regression coefficients [55]. Whenever not set in the default, the methods were set to use microbial taxa with more than 10% prevalence across samples and centered-log ratio (CLR) transformation. Microbial taxa that had *P* [ALDEx2 (wi.eBH), ANCOM-BC, and LinDA] or *Q* (MaAslin2) values ≤ 0.05 after false discovery rate correction were considered significant and added to the network analysis. The taxa significant in each method were fed to the ensemble method. A final network analysis was performed to identify the microbial taxa that was consistently significant across different statistical tests; this way, less noise and a greater level of relevance could be achieved amongst identified different microorganisms. A final correlation of the identified microbial taxa was performed with the trait of interest using the extreme phenotype cows to express the positive or negative correlation of the microbial taxa with the trait.

### Methane production, yield, and intensity calculations

An analysis was performed investigating the selection of the most efficient cows instead of the least efficient ones using feed and milk production efficiency strategies and their effects on the daily performance and carbon footprint of current lactating dairy cows. Analyses were performed using the extremes of each group (least efficient: *n* = 50; most efficient: *n* = 50) to resemble the potential differences we would find in carbon footprint reduction using these methods. Methane production (g/d per cow) was assessed to all 454 cows based on the predictive model (Eq. 27) from Nielsen, et al. [56] validated for cows in North America [57]. The equation was as follow:

CH_4_ (g/d per cow) = ([1.23 x DMI (kg/d)– 1.45 x dietary fatty acid content (% of DM) + 0.120 x neutral detergent fiber content (**NDF**; % of DMI)]/0.05565)

The corrections for CH_4_ yield (g/kg DMI) and intensity (g/kg NESec) were calculated based on measured production traits. A correction of CH_4_ production (g/d) for gross feed efficiency (**GFE**; g of milk produced per g DMI) was performed to access the CH_4_ production reduction potential when considering both CH_4_ yield and intensity. Then, all 454 cows were ranked from most to least RFI efficiency status, and the top 50 most efficient cows were compared to the bottom 50 least efficient ones when these were ranked based on the measured RFI, microbiome predicted RFI, and genomic PTA RFI.

## Results

### Potential rumen microbiome contribution to residual feed intake

**Fig. 2:**
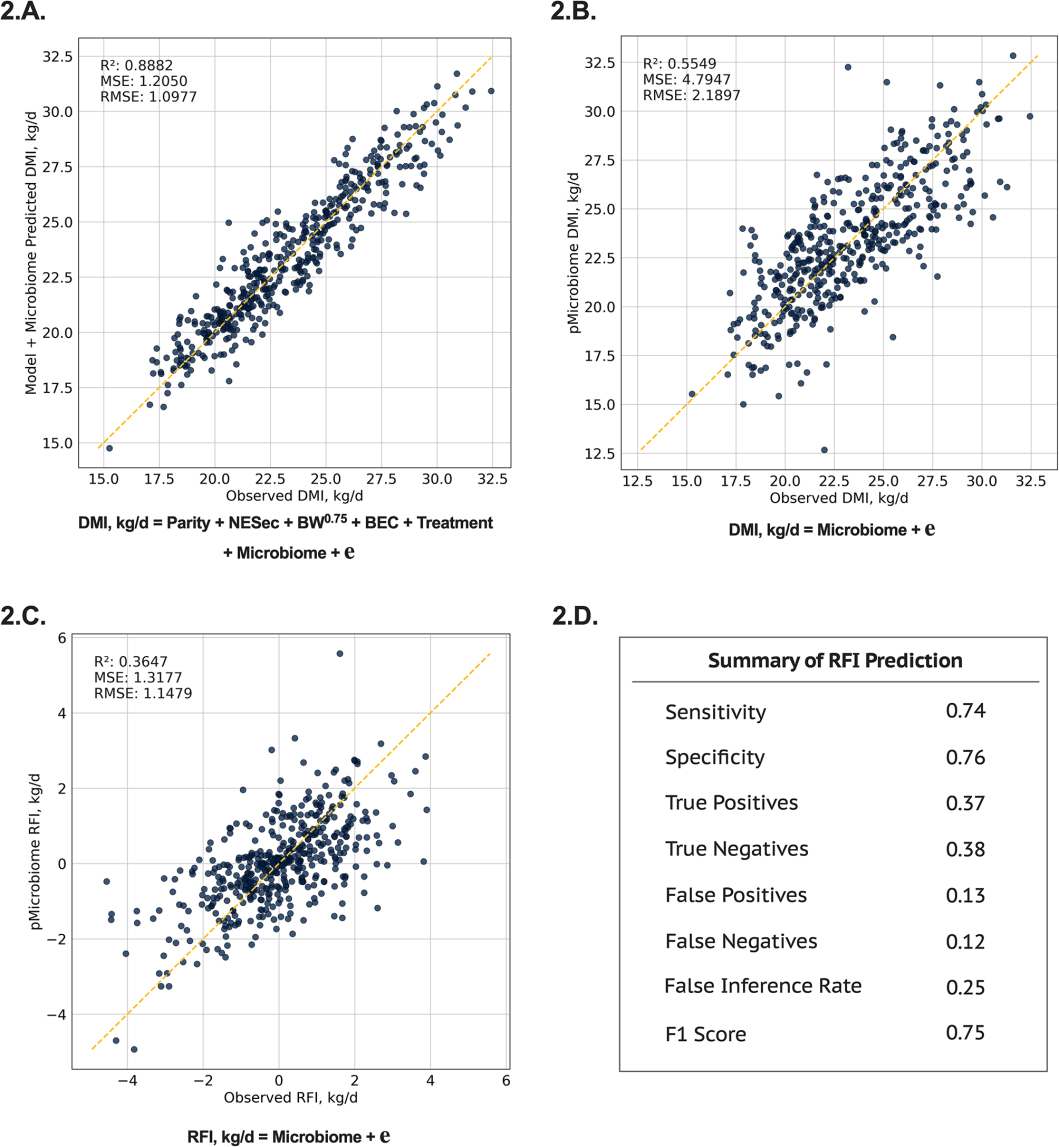
Machine learning prediction using feature selection and Ridge regression with 10-fold cross-validation on the residual feed intake (**RFI**) of lactating dairy cows. **2.A.** Model used for the prediction of dry matter intake (**DMI**) containing the main energy sinks in a lactating dairy cow (NESec, MBW, and BEC), the effect of parity, the effect of previous treatment plus the effect of the rumen microbiome; **2.B.** Prediction of DMI using only the rumen microbiome (pMicrobiome) to explore the overall contribution of the rumen microbes to feed intake; **2.C.** Prediction of the residual DMI, also known as RFI, using only the rumen microbiome composition; and **2.D.** Summary of RFI prediction with the rumen microbiome, from which confusion matrix was derived from comparing extracted observed vs. predicted residuals. The loss function for the AI model was the mean squared error (**MSE**) and the scoring metric was R^2^

**Fig. 3:**
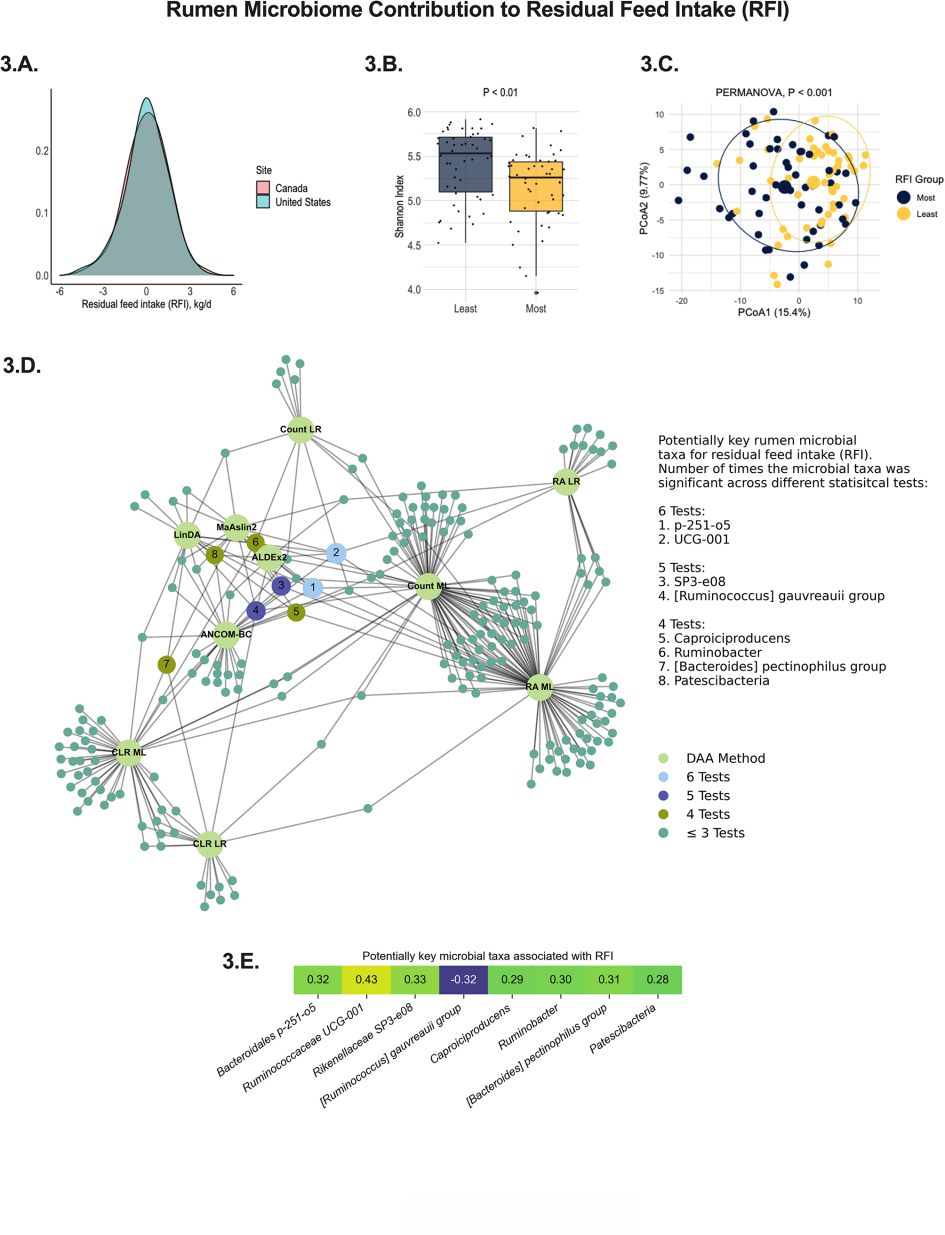
An ensemble method to depict the rumen microbiome signature of residual feed intake **(RFI)** in lactating dairy cows. The method consisted of summarizing the differential abundance analyses of some of the most robust methods available in the literature. **3.A.** Distribution of RFI means among dairy cows in the studied cohort depicting differences between US and Canadian cows. **3.B.** Shannon index, as a representation of alpha-diversity differences between the extremes of least and most efficient cows of the RFI phenotype, showing that the rumen microbial community diversity is lower among the most efficient cows. **3.C.** Principal coordinate analysis (**PCoA**) of the rumen microbiome showing that the extremes of least and most efficient cows of the RFI phenotype in the study have a set of different microorganisms that could potentially modulate this trait. **3.D.** Summary of all statistically significant microbial taxa (*P* < 0.05) in the RFI phenotype that have greater potential to not be affected by the sources of variation evaluated in the study. Ensemble analysis was performed using a STEPWISE selection (lowest AICC) in linear regression analysis (microbial taxa tested as microbial count, relative abundance, and centered-log ratio), machine learning regression (microbial taxa tested as microbial count, relative abundance, and centered-log ratio), and four independent differential abundance analyses tests: ALDEx2, ANCOM-BC, MAaslin2, and LinDA (contrast of phenotypic extremes; least efficient *n* = 50 cows vs. most efficient *n* = 50 cows). Microorganisms are ranked according to their significant prevalence across tests and represent potential key microbial taxa contributing to the trait of interest. **3.E.** Correlation between normalized (CLR) key microbial players and RFI in the extreme population of least and most efficient cows. Only microbial taxa different in at least 4 statistical tests are displayed in these results

The rumen microbiome improved DMI prediction when integrated with standard variables used in DMI predictive equations (R^2^ = 0.89, Fig. 2.A.). When used to predict DMI and feed efficiency, the rumen microbiome composition explained a significant portion of DMI (R^2^ = 0.55, Fig. 2.B.) and RFI (R^2^ = 0.36, Fig. 2.C) variations. After performing the prediction of RFI based on the rumen microbiome, all cows were ranked based on the microbiome predicted RFI and classified into two groups: positive (not efficient) and negative (efficient) RFI status. The model harmonic mean of precision and true positive rate (F1 Score) shows the prediction was 75% accurate at discriminating whether a cow was more or less feed efficient (Fig. 2.D.). Regarding the variation in RFI that could be potentially modulated by the rumen microbiome, we further conducted a network analysis of ensemble differential abundance methods to identify key microbial players linked to differences in the core microbiome (microbes statistically associated with the trait in at least four statistical tests) (Fig. 3). Consequently, alpha and beta-diversity indexes differed among RFI groups in the extreme of feed efficiency (Fig. 3.B. and 3.C.). From those, the Shannon index that provides more robust information for the sample distribution regarding microbial richness and evenness was lower in most efficient cows when compared to least efficient ones (5.26 vs. 5.55; *P* < 0.01). Our ensemble networks analysis showed that when contrasting microbial differences in RFI phenotype extremes and expanding it to the total population, a total of 8 microbes were deemed consistently different in at least 4 statistical tests, indicating that these microbial taxa might be important players for cows in the RFI extremes for the overall population of the study. Of those 8 microbes, most had a positive relationship with RFI, and only *[Ruminococcus] gauvreauii group*, that is, from the *Lachnospiraceae* family, was negatively correlated, which means only this last genus was positively associated with an improved efficiency of the cow (a negative RFI represents a cow had a lower DMI intake than expected).

### Potential rumen microbiome contribution to gross feed efficiency measures

**Fig. 4:**
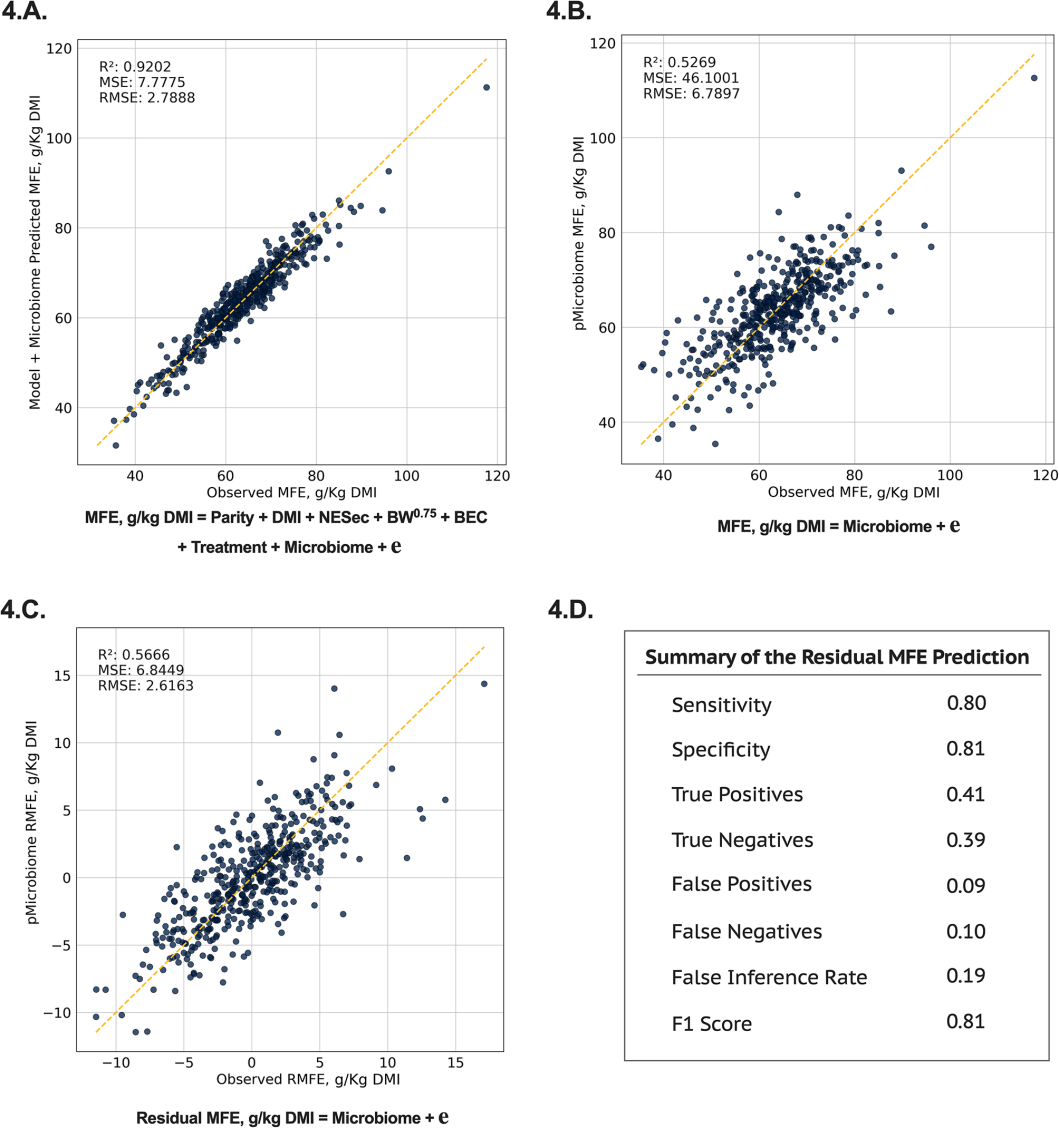
Machine learning prediction using feature selection and Ridge regression in 10-fold cross-validation on the milk fat efficiency (**MFE**) of lactating dairy cows. **4.A.** Model used for the prediction of MFE containing the main energy input and sinks in a lactating dairy cow (DMI, NESec, MBW, and BEC), the effect of parity, the effect of previous treatment plus the effect of the rumen microbiome; **4.B.** Prediction of MFE using only the rumen microbiome to explore the overall contribution of the rumen microbes to the trait; **4.C.** Prediction of the residual MFE, using only the rumen microbiome composition to depict the contribution of the rumen microbiome to the unexplained variance in the trait; and **4.D.** Summary of the residual MFE prediction with the rumen microbiome, from which confusion matrix was derived from comparing extracted observed vs. predicted residuals. The loss function for the AI model was the mean squared error (**MSE**), and the scoring metric was R^2^

**Fig. 5:**
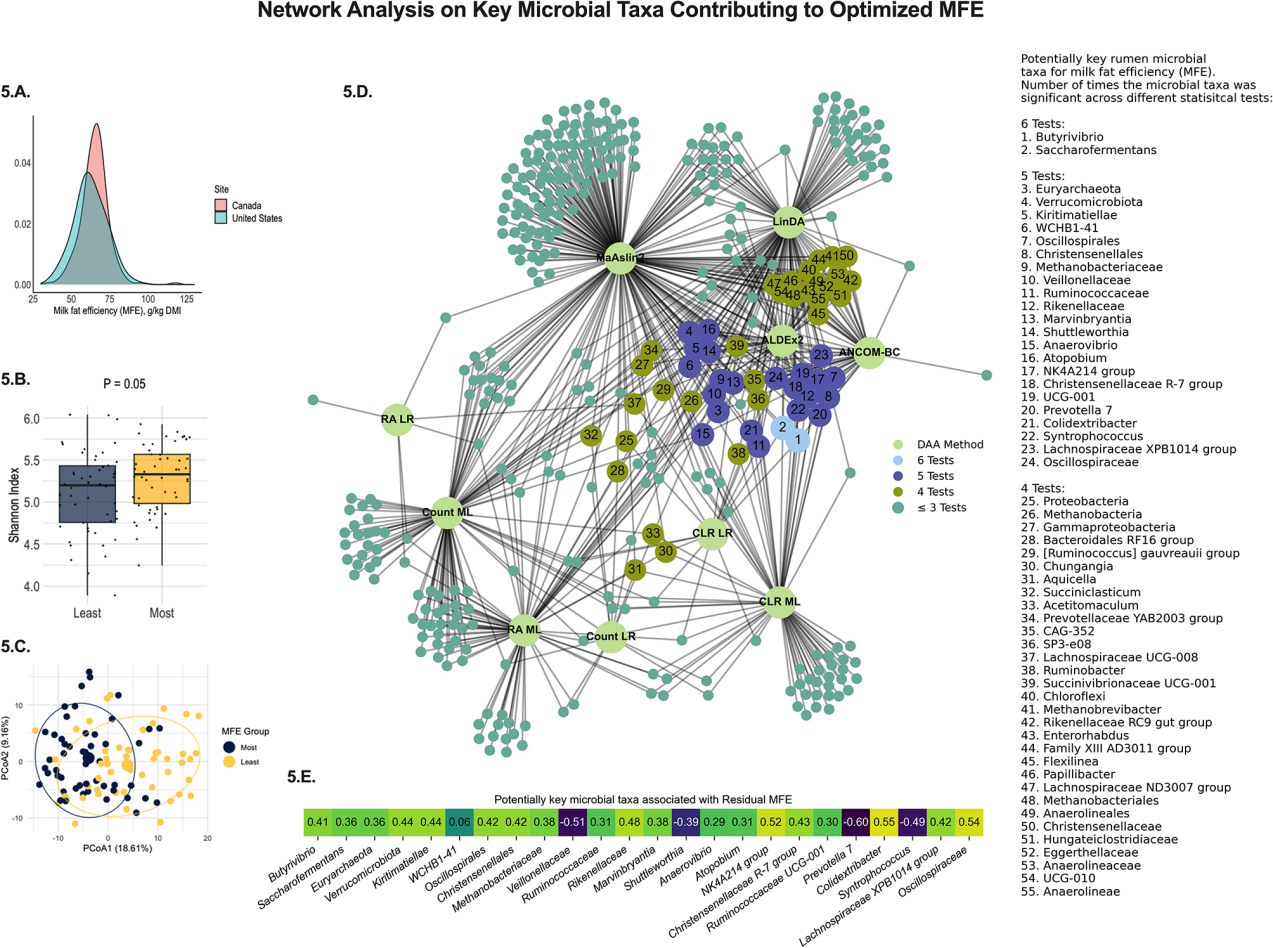
An ensemble method to depict the rumen microbiome signature for milk fat production efficiency (**MFE**). **5.A.** Distribution of MFE means among dairy cows in the studied cohort depicting differences between US and Canadian cows. **5.B.** Shannon index, as a representation of alpha-diversity differences between extremes of least and most efficient cows of the MFE phenotype, showing that the rumen microbial community diversity is higher among the most efficient cows. **5.C.** Principal coordinate analysis (**PCoA**) of the rumen microbiome showing that the extremes of least and most efficient cows of the MFE phenotype in the study have a set of different microorganisms that could potentially modulate this trait. **5.D.** Summary of all statistically significant microbial taxa (*P* < 0.05) in the residual MFE phenotype that have greater potential to not be affected by the sources of variation evaluated in the study. Ensemble analysis was performed using a STEPWISE selection (lowest AICC) in linear regression analysis (microbial taxa tested as microbial count, relative abundance, and centered-log ratio), machine learning regression (microbial taxa tested as microbial count, relative abundance, and centered-log ratio), and four independent differential abundance analyses tests: ALDEx2, ANCOM-BC, MAaslin2, and LinDA (contrast of phenotypic extremes; least efficient *n* = 50 cows vs. most efficient *n* = 50 cows). Microorganisms are ranked according to their significant prevalence across tests and represent potential key microbial taxa contributing to the trait of interest. **5.E.** Correlation between normalized (CLR) key microbial players and residual MFE in the extreme population of least and most efficient cows. Only microbial taxa different in at least 4 statistical tests are displayed in these results

**Fig. 6:**
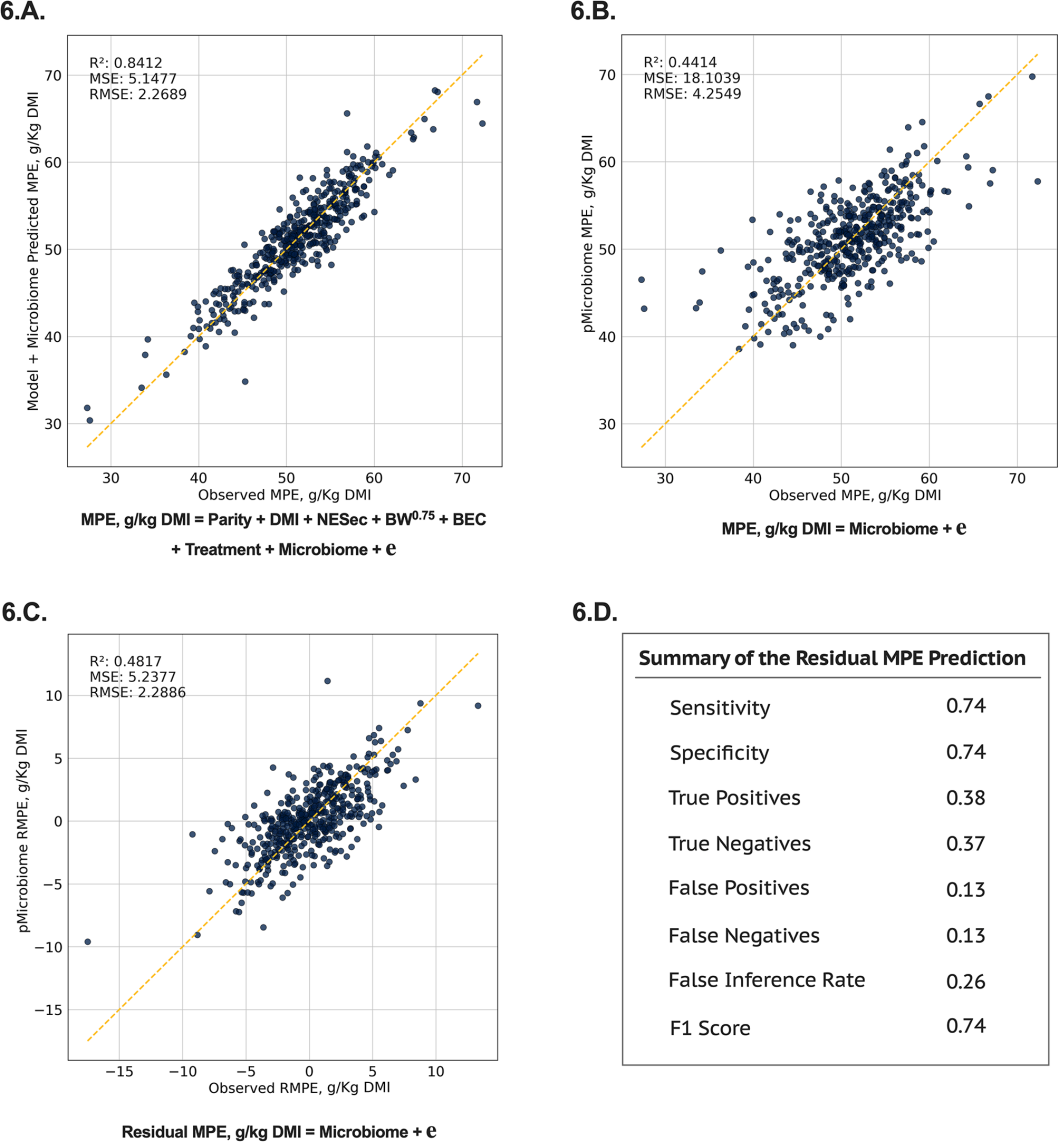
Machine learning prediction using feature selection and Ridge regression in 10-fold cross-validation on the milk protein efficiency (**MPE**) of lactating dairy cows. **6.A.** Model used for the prediction of MPE containing the main energy input and sinks in a lactating dairy cow (DMI, NESec, MBW, and BEC), the effect of parity, the effect of previous treatment plus the effect of the rumen microbiome; **6.B.** Prediction of MPE using only the rumen microbiome to explore the overall contribution of the rumen microbes to the trait; **6.C.** Prediction of the residual MPE, using only the rumen microbiome composition to depict the contribution of the rumen microbiome to the unexplained variance in the trait; and **6.D.** Summary of the residual MPE prediction with the rumen microbiome, from which confusion matrix was derived from comparing extracted observed vs. predicted residuals. The loss function for the AI model was the mean squared error (**MSE**) and the scoring metric was R^2^

**Fig. 7:**
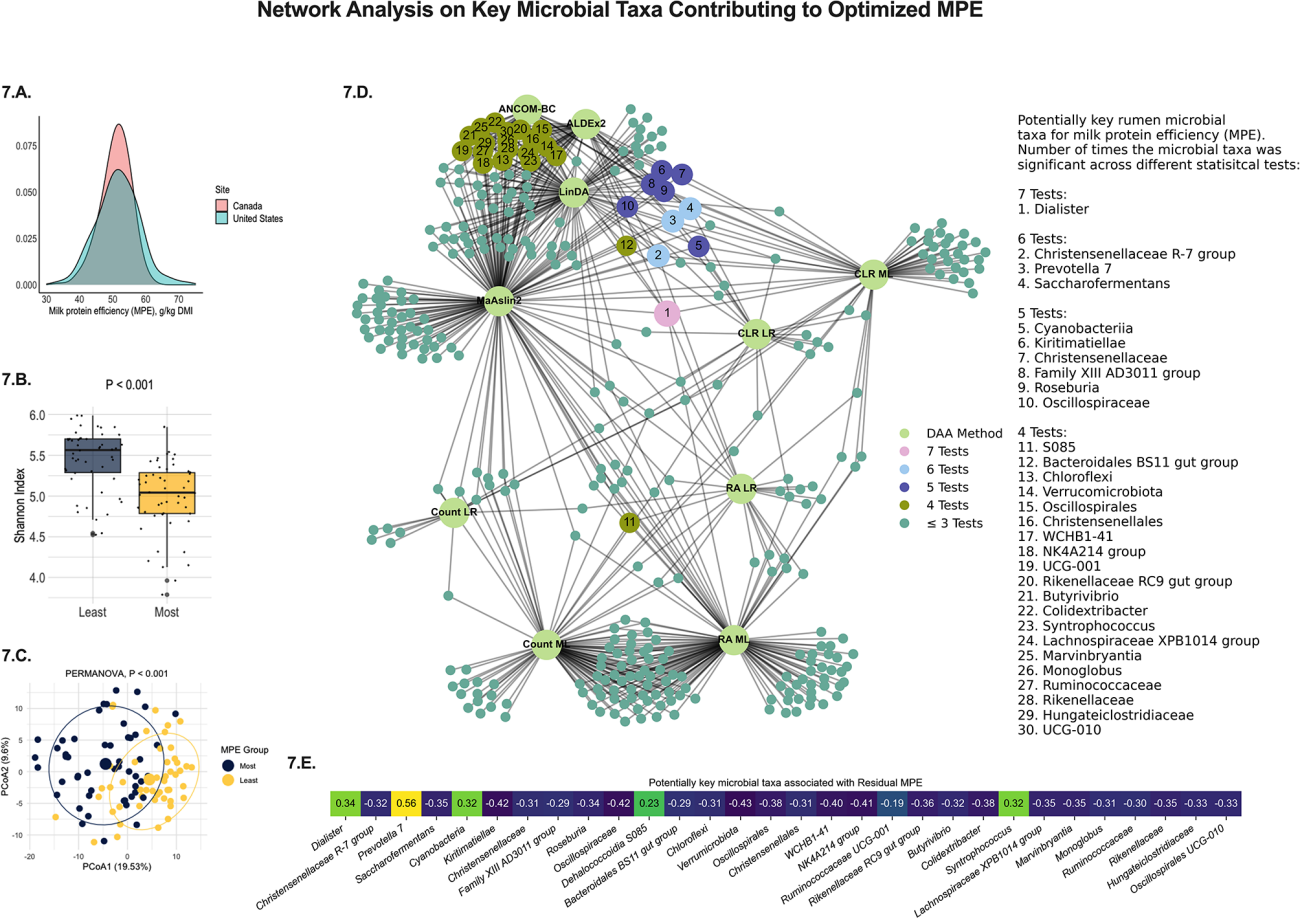
An ensemble method to depict the rumen microbiome signature for milk protein production efficiency (**MPE**). **7.A.** Distribution of MPE means among dairy cows in the studied cohort depicting differences between US and Canada cows. **7.B.** Shannon index, as a representation of alpha-diversity differences between extremes of least and most efficient cows of the MPE phenotype, showing that the rumen microbial community diversity is lower among the most efficient cows. **7.C.** Principal coordinate analysis (**PCoA**) of the rumen microbiome showing that the extremes of least and most efficient cows of the MPE phenotype in the study have a set of different microorganisms that could potentially modulate this trait. **7.D.** Summary of all statistically significant microbial taxa (*P* < 0.05) in the residual MPE phenotype that have greater potential to not be affected by the sources of variation evaluated in the study. Ensemble analysis was performed using a STEPWISE selection (lowest AICC) in linear regression analysis (microbial taxa tested as microbial count, relative abundance, and centered-log ratio), machine learning regression (microbial taxa tested as microbial count, relative abundance, and centered-log ratio), and four independent differential abundance analyses tests: ALDEx2, ANCOM-BC, MAaslin2, and LinDA (contrast of phenotypic extremes; least efficient *n* = 50 cows vs. most efficient *n* = 50 cows). Microorganisms are ranked according to their significant prevalence across tests and represent potential key microbial taxa contributing to the trait of interest. **7.E.** Correlation between normalized (CLR) key microbial players and residual MPE in the extreme population of least and most efficient cows. Only microbial taxa different in at least 4 statistical tests are displayed in these results

The potential rumen microbiome contribution to the efficiency to produce milk fat (Fig. 4 and 5) and milk protein (Fig. 6 and 7) are shown below. Integrating the rumen microbiome with the known sources of variation of gross feed efficiency measures also improved the prediction of milk fat production efficiency (**MFE**; R^2^ = 0.92, Fig. 4.A.) and milk protein production efficiency (**MPE**; R^2^ = 0.84, Fig. 6.A.). Similar to RFI, the rumen microbiome explained most of the variation in MFE and MPE (R^2^ = 0.53 and 0.44, respectively, Fig. 4.B. and 6.B.). Given that MFE and MPE are highly modulated by the level of net energy secreted in milk (**NESec**) and DMI level (Table 1), these findings reinforce the hypothesis that the rumen microbiome may contribute to the modulation of some traits previously documented to be modulated by host genetics [2, 9]. To avoid this possible host-microbiome confounding effect on the modulation of MFE and MPE, we aimed at isolating these effects and investigated the residuals of MFE and MPE instead, as these would have a greater potential to be primarily modulated by the rumen microbiome. Our analysis showed that from the 16 and 31% of residuals in MFE and MPE (Table 1), respectively, the rumen microbiome explains 57 and 48% of that variation (Fig. 4.C. and 6.C.). When evaluating the precision and accuracy for the prediction of these residuals, the predictive models were good at discriminating whether a cow was more or less efficient in producing milk fat and protein beyond what is modulated by those known sources of variation, such as DMI and NESec (F1 Score: residual MFE = 0.81 and residual MPE = 0.74, Fig. 4.D. and 6.D.).

Different than RFI, the core microbial community of these potentially modulating portions (residuals) of MFE and MPE were highly complex (Figs. 5 and 7). For the residual MFE, 55 microbial taxa were deemed to be the core microbiome of this trait, as they were found to be important to the trait in 4 or more statistical analyses used in this study. In this scenario, the genera *Butyrivibrio* and *Saccharofermentans* were deemed key microbial players from the core microbiome as these were significantly different in more analysis than all other microorganisms. Overall, most genera in the core microbiome were positively correlated with MFE. For the residual MPE, 30 microbial taxa were deemed to be the core microbiome of this trait. The key microbial players from the core microbiome in this case were *Christensenellceae R-7 group*, *Prevotella 7*, and *Saccharofermentans* genera, that were different in 6 differential analyses, and *Dialister*, which was in 7 analyses. Interestingly, most genera in this case were negatively correlated with MPE, which is the opposite that was found to MFE. These relationships may be because residuals of MFE and MPE have a -71.3% correlation, suggesting an increase in the production efficiency of one trait mediated by the microbiome may be at the expense of the other milk component’s efficiency. On the other hand, RFI had a 0.1% and 0.2% correlation with residual MFE and residual MPE, respectively, which may explain the distinct microbes associated with RFI. Furthermore, we observed the almost negligible correlation of RFI with residual MFE and MPE was a consequence of the correction of these residuals to observed DMI, since DMI was considered in the model. Contrarily, RFI was a residual derived from the difference between predicted and observed DMI, thus, this residual is a consequence of correcting for the parameters in the model (Parity, MBW BEC, NESec, and cohort) but not DMI level. Overall, these findings suggest that microbes associated with the residuals of MFE and MPE are likely not a consequence of feed intake level and is more closely related to specialized end-product supply to the host, while those associated with RFI can be directly or indirectly associated with a digestion efficiency that changes feed intake level.

### Potential effects on carbon footprint when selecting more feed efficient cows

**Fig. 8:**
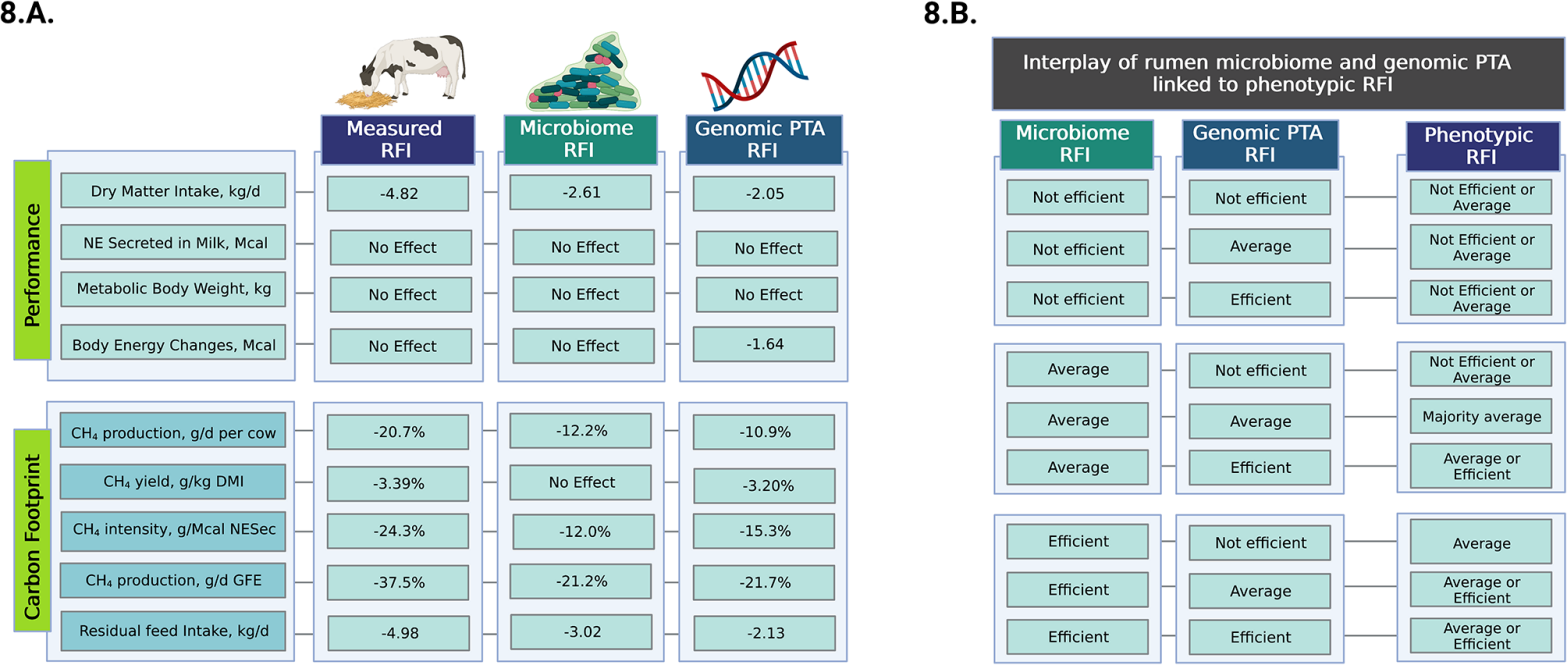
Hypothetical selection of feed efficient cows for residual feed intake (**RFI**) and the interplay of the rumen microbiome, genomic predicted transmitting ability (**PTA**), and phenotypic RFI. **8.A.** Comparison of daily performance and carbon footprint between the most efficient cows (*n* = 50) and the least efficient cows (*n* = 50) based on residual feed intake and gross feed efficiency. Methane production (g/d per cow) was assessed based on the predictive model (Eq. 27) from Nielsen, et al. (70) validated for cows in North America (71). The equation was as follow: CH_4_ (g/d per cow) = ([1.23 x DMI (kg/d)– 1.45 x dietary fatty acid content (% of DM) + 0.120 x neutral detergent fiber content (NDF; % of DMI)]/0.05565). The corrections for CH_4_ yield (g/kg DMI) and intensity (g/kg NESec) were calculated based on measured production traits. A correction of CH_4_ (g/d) for gross feed efficiency (GFE; g of milk produced per g DMI) was performed to access the CH_4_ production reduction potential when considering both CH_4_ yield and intensity. Numeric values are significant differences in selecting the most efficient cows when *P* ≤ 0.05; Methane results, including group means, SEM, and *P*-values are shown in Supplementary Table 2. **8.B.** Outcome of the rumen microbiome and genomic PTA predictions for RFI showing their potential interplay in determining the phenotypic RFI of the studied lactating dairy cows

A hypothetical cow selection analysis performed on the evaluated feed and milk production indexes indicates that selection for RFI is a promising approach to reducing carbon footprint of dairy cows (Fig. 8.A.). Besides not affecting daily cows’ lactation performance, metabolic body weight, and body energy changes, the selection based on extreme RFI animals has the potential to decrease almost 5 kg of diet DM that is consumed by each cow per day, based on the measured phenotypic RFI (Fig. 8.A.). Furthermore, an absolute decrease of 20.7% of daily CH_4_ produced was observed to be possible, while CH_4_ production corrected for yield (g/kg DMI) and intensity (g/Mcal NESec) could have a reduction of 37.5% when selecting the most efficient cows in the cohort. Selection for the most efficient cows based on rumen microbiome and genomic PTA predictions of RFI decreased CH_4_ production at about half of the potential reduction from using directly measured RFI. The use of the residual for MFE and MPE did not affect the daily cow’s performance, RFI, or carbon footprint. Finally, the outcomes of the rumen microbiome and genomic PTA predictions for RFI interplay were explored by splitting the dataset into three categories (efficient, average, and not efficient; Fig. 8.B.). Prediction outcomes revealed that in order for a cow to obtain an efficient RFI, a combination of the either average and efficient microbiome and genomic PTA prediction for RFI was needed. Whenever the microbiome or genomic PTA prediction of RFI of the cow was defined as not efficient RFI, then phenotypic RFI was never efficient.

## Discussion

These results reinforce the possibility that other non-genetic factors should be identified to better retain feed efficient cows in a herd. In this context, the study highlights the importance of the rumen microbiome as a phenotypic trait of lactating dairy cows. Long pointed to play a major role in the supply of energy, protein, vitamins, and bioactive compounds to the host [16–18], the rumen microbiome in our study considerably improved DMI prediction when integrated with previous standard variables or even alone. These findings suggest that the unexplained variance in DMI found with linear mixed models (RFI index; Table 2) comes in part from the rumen microbiome composition, which promotes the degradation of dietary nutrients and supply of end-products of fermentation to dairy cows [58]. Given that the rumen microbiome composition explained most of the variation in DMI, which is highly heritable by host-genetics [59], there might be certain traits in ruminants that could be modulated by an inter-play between the rumen microbiome and host-genetics instead. The findings from this study also shows that the rumen microbiome composition explained 36% of RFI variation, which makes it a major source of variation for this feed efficiency trait. The difference in the coefficient of determinations (R^2^) between the linear mixed model DMI regression (R^2^ = 0.81), the RFI prediction with the rumen microbiome (R^2^ = 0.36), and the full DMI prediction model considering all of these variables (R^2^ = 0.89) also suggests an interaction of the rumen microbiome with DMI predictive variables that further contributed to the explained variability of the model.

Besides accounting for 36% of the variation in RFI, the rumen microbiome was 75% accurate at indicating whether a cow was ranked according to their least or most efficiency status. The reliability of RFI prediction is a current challenge in genetic selection [15]; however, the consistency of RFI during and across lactation, and of the rumen microbiome from at least early to mid-lactation of dairy cows [25, 30, 60] suggest that the microbiome can be a tool to improve the prediction of traits currently only performed with genomic information. Although rumen microbiome assessment is not feasible in large scale, proxies such as through buccal, nasal, and fecal microbiomes, or even blood, urine, and milk biomarkers could be alternatives to capture gastrointestinal contribution to RFI phenotypic variation [25, 61]. Another alternative beyond genetic selection would be to provide an early efficiency assessment per lactation for these cows so better grouping strategies could be elaborated based on their efficiency statuses. Grouping cows into more homogenous RFI groups, especially based on feed and milk production efficiency, could have a profound impact on reducing dietary nutrient waste (thus, the carbon footprint) and feed costs in dairy farms, as previously reported to other production traits [62, 63]. If less feed efficient cows can be identified, dietary modification strategies, such as through supply of microorganisms associated with improved efficiency or supplementation of bioactive molecules protected from rumen degradation, might become a possibility to improve their efficiency status.

The lower alpha-diversity indexes represented here through Shannon index show that, the more efficient cows have a more homogenous and less diverse microbiome community composition than those least efficient one’s, as previously reported [21]. The decrease in microbial diversity might represent a more consistent microbiome that is specialized in producing key microbial enzymes to degrade complex nutrients (e.g., CA-zymes) [64, 65]. In a previous study evaluating the enzyme activity of rumen microbes from Angus bulls diverging in feed efficiency [64], more efficient animals were reported to have greater relative abundance of key ruminal CAZ-zymes, such as endo-β- 1,4-xylanase, endoglucanase, and carbohydrate-binding modules (CBMs) involved in cellulose degradation beyond of a greater capacity to utilize cellulose and xylose. Furthermore, feed efficient animals in the aforementioned study also had greater relative abundance of ammonia assimilation functions in rumen microbes, suggesting that at least the rumen of more efficient beef cattle have more specialized enzymes for lignocellulosic biomass degradation and utilization beyond of an increased demand for nitrogen assimilation. Interestingly, in lambs, the *[Ruminococcus] gauvreauii group* that was a major microbe associated with improved RFI, has been reported to have a strong association with total ruminal volatile fatty acids concentration, specifically acetate, propionate, butyrate, and valerate, and to be positively associated with rumen microbial protein synthesis [66], which support the hypothesis of less diverse yet more efficient rumen microbiome. In environments outside ruminants, a study has reported that isolates of *[Ruminococcus] gauvreauii group* from human bile could later be cultured under end-products of starch fermentation, especially formate, while producing acetate [67], showing these microbes coming from such conditions may also employ the Wood Ljungdahl pathway. Despite the difference in environments, the findings from that study and our study deserves further investigation given that formate is a major substrate linked to methanogenesis [68], and perhaps this microbe may turn metabolites that would otherwise be wasted during ruminal fermentation (e.g., formate as a source for CH_4_ synthesis) into valuable precursors of milk synthesis. Overall, these results indicate that the observed microbial differences may be another indication of improved fiber fermentation and rumen nitrogen metabolism in the most efficient cows. Despite the interesting potential of each microbe in changing ruminal fermentation, the moderate correlation of these key microbial players suggests the collective contribution of different microorganisms to RFI is a critical factor determining their association with the trait instead of their independent contribution.

When evaluating the association of the rumen microbiome composition with gross efficiency to produce milk fat, most genera in the core microbiome were positively correlated with the residual MFE, and contrary to RFI, most efficient cows had an increase in microbial alpha-diversity. Given that acetic acid is the most common end-product of fermentation in the rumen and a major contributor to milk fat synthesis in dairy cows [18, 68], the increase in alpha-diversity may represent a greater likelihood of increasing microbes that are generalists and produce acetate, increasing the capacity to produce milk fat from the fermented nutrients. The greater abundance of *Butyrivibrio* in more MFE cows may be a further example of the increase in alpha-diversity. Using metatranscriptomics [65], a study has recently reported that this genus is one of the major bacteria in the rumen to produce enzymes that degrade degradable fiber (cellulose, hemicellulose, and pectin) and starch, suggesting greater *Butyrivibrio* abundance may be associated with cross-feeding. Furthermore, the main short-chain fatty acid from *Butyrivibrio* fermentation is butyric acids, which together with acetic acid are the major end-products of fibrolytic fermentation and primary precursors of milk fat synthesis [16, 18, 68]. Thus, the positive linear association of rumen *Butyrivibrio* with cows that are more efficient in producing milk fat, along with the greater spectrum of microorganisms growing in the rumen of these cows, suggest that *Butyrivibrio* might be key organisms to MFE. Studies employing more detailed approaches (e.g., shotgun sequencing and metatranscriptomics) would contribute by evaluating which species or even genomes within *Butyrivibrio* and other highlighted genera may be exactly contributing to these effects, as these genera has vast genetic diversity.

In regard to MPE, the diversity followed a similar distribution as that observed in RFI groups, which alpha-diversity was lower in the rumen of the most efficient cows. Milk protein synthesis is associated with the volume of milk produced (*r* = 0.91 in the current study) [69]; thus, there is a possibility that the decrease in rumen microbial diversity in most efficient cows represents a dominance of microorganisms that more closely produce gluconeogenic precursors for milk synthesis, such as propionic acid-producing bacteria. In the current study, the dominance variable that measures the abundance of most abundant microbial taxa was greater for most efficient cows (*P* < 0.05), suggesting that a more homogenous and less diverse ruminal microbial fermentation may once again be the key to more efficient microbiomes. In ruminants, the Firmicutes genera *Dialister* that was one of the main organisms associated with MPE efficiency and in greater concentration in most efficient cows, have also been reported to be positively associated with increased milk yield [23] and milk production efficiency [70] in previous studies. One potential explanation is that rumen *Dialister* in sheep has been reported to be positively associated with total ruminal volatile fatty acids and negatively associated with lactic acid and lipopolysaccharides (LPS), which suggests positive association with fermentation efficiency and improved ruminal health [71, 72]. Another possibility to explain this association is that rumen microbes may produce bioactive metabolites with the potential to modulate the cow’s physiology, such as tryptophan derivatives and other metabolites with the neuromodulation potential, gut barrier integrity, hormonal changes, and others explored in human gut microbiome studies [73]. Considering the rumen microbiome is strategically placed in the cow prior to their main absorption sites, and so bioactive metabolites have even greater chances to be absorbed in cows than in humans, there is a vast field of bioactive metabolite exploration in dairy cows and ruminants overall that deserves further investigation.

Interestingly, *Prevotella 7* was one of the most associated with both MFE (*r* = -0.60) and MPE (*r* = 0.56), but in opposite ways. The opposite relationship of microbes and efficiency traits was also observed for *Butyrivibrio* and *Saccharofermentans*, which were positively associated with MFE but negatively associated with MPE. *Prevotella* has long been reported to have a broad enzymatic capacity and to be one of the main proteolytic organisms in the rumen. However, recent metagenomics deep sequencing analysis shows that rumen *Prevotella* in goats are also some of the most active lignocellulosic organisms, with the potential to degrade starch, cellulose, hemicellulose, and pectin [74]. In the entire population of this study, the abundance of *Prevotella* was negatively associated with both *Butyrivibrio* (*r* = -0.44) and *Saccharofermentans* (*r* = -0.23), suggesting the dominance of *Prevotella* may bring benefits to overall dietary nutrient digestion and milk synthesis when compared to more fiber degrading microbes as *Butyrivibrio*. Important to note, the function of organisms within these genera can be highly diverse, suggesting the exploration of these results with species or even genome-level approaches (e.g., shotgun metagenomics and metatranscriptomics) is fundamental to elucidate the mechanisms utilized by these microbes.

These results also highlight the genetic selection for RFI as a critical determinant for the carbon footprint of the dairy sector. By showcasing that selection based on RFI has the potential to significantly reduce diet consumption without compromising vital parameters like lactation performance, the study opens avenues for more sustainable dairy farming practices, which is currently needed in ruminant production systems [5]. By taking advantage of traits that are would not affect the long-term productivity and health of dairy cows, as RFI and the residuals of MFE and MPE that were used in this study and are less prone to be affected by the levels of DMI, NESec, MBW, BEC, and Parity, greater are the chances of holistically securing the sustainability goals of the sector. Despite mixed results in previous studies evaluating methane emissions in cows differing in RFI [6, 75], the substantial potential decline in daily CH_4_ production in a large population of dairy cows from this work suggests selection of animals with a lower carbon footprint is likely viable and can contribute to global efforts in methane reduction previously described per the Paris Agreement [7]. The potential synergistic role of the rumen microbiome and host genetics in determining the phenotypic RFI also reinforces the multifaceted nature of feed efficiency. This research underscores the need for a holistic approach for the use of feed efficiency measures in the field in order to improve milk production and dairy farm sustainability. By integrating genomic information with the methodologies of artificial intelligence and bioinformatics and synergizing them with contemporary findings in microbiology and ruminant nutrition or even biomarkers that can improve the predictability of these traits, our research proposes a new overview to handle complex endeavors in this trajectory of advancing the dairy sector.

## Conclusions

In summary, by using an artificial intelligence approach, this study shows that the rumen microbiome composition explains a significant portion of the variation in RFI, presenting a promising site of exploration for future improvements in predictive models to decrease the dairy sector’s carbon footprint. The associations of RFI as well as MFE, MPE, and their residuals with the rumen microbiome, unraveled through an ensemble method, further indicates key microbial players that could be targeted to further evaluate their effect on the efficiency of dairy cows. Additionally, the predictability of heritable traits by the rumen microbiome underscores the need for future research to dissect host-microbiome interactions in shaping feed and milk production efficiency. This exploration, and consequently further validation studies with complementary results from digestive parameters (e.g., digestibility) to more detailed microbiome approaches (shotgun metagenomics, metatranscriptomics, and metabolomics), is vital to pioneer advances in ruminant nutrition and fortify sustainable dairy production pathways.

### Electronic supplementary material

Below is the link to the electronic supplementary material.

Supplementary Material 1

Supplementary Material 2

## Data Availability

All rumen microbiome sequences of this study are current available on the Sequence Read Archive of the National Center for Biotechnology Information under the BioProject accession number PRJNA962991. Scripts from this work can be accessed on https://github.com/hugofmonteiro/rumen-microbiome-feed-efficiency.
